# Perception and usage of short-term prednisone and prednisolone in dogs

**DOI:** 10.1186/s12917-023-03644-x

**Published:** 2023-07-24

**Authors:** Margaret Gober, Andrew Hillier

**Affiliations:** Zoetis LLC, 10 Sylvan Way, Parsippany, NJ 07054 USA

**Keywords:** Corticosteroids, Glucocorticoids, Side effects, Dogs, Prednisone, Prednisolone, Allergic skin disease

## Abstract

**Background:**

Corticosteroids are widely used with low rates of reported side effects and a broad level of comfort in the hands of most veterinarians. With a low side effect reporting level of < 5% and high level of comfort there may be complacency and underestimation of the impact side effects of corticosteroids may have on a pet and pet owner.

**Objective:**

The objective of this clinical study was to describe the experience and perception of an owner who administered anti-inflammatory doses of oral prednisolone and prednisone to their dog for up to 14 days. We hypothesized dogs receiving anti-inflammatory doses of prednisone and prednisolone would experience much greater rates of side effects by day 14 then reported in current literature.

**Animals:**

There were 45 dogs initially enrolled in the study.

**Results:**

At each study point, 31 owners provided results. On day 5, 74% (23/31) reported at least 1 change in their dog’s behavior including polyuria, polydipsia, polyphagia, polypnea and/or increased vocalization, with 11 individuals (35%) reporting these changes greatly increased. On day 14, 90% of owners (28/31) reported at least 1 change in their dog’s behavior including polyuria, polydipsia, polyphagia, and/or polypnea as the most common changes noted. Overall, 61% (19/31) of owners reported an increase in filling of the water bowl over baseline and one-third (11/31) of pet owners reported cleaning up urinary accidents for pets who had been continent prior to the start of the study. Pet owner steroid satisfaction remained high through day 14 at 4.5/5 (1 = very unsatisfied, 5 = very satisfied).

**Conclusion:**

This study highlights the impact short term anti-inflammatory doses of prednisone or prednisolone have on dog behaviour and confirms our hypothesis that by day 14, 90% of dogs experienced one or more behaviour changes, with polyuria and polydipsia most commonly reported. Adverse events were noted regardless of starting dosage or regimen. Although most pet owners expressed satisfaction with steroid treatment due to its high efficacy, 70% would select a more costly treatment if that treatment had fewer side effects.

## Introduction

Steroids (glucocorticoids/corticosteroids) have been one of the most commonly prescribed medications in veterinary medicine since the 1950’s, and while the reasons for use may have changed, they continue to be a common pharmacological intervention [1, 2]. Corticosteroids are used for many reasons and are commonly used for the treatment of canine dermatitis [2, 3]. While it is estimated 3–15% of dogs have atopic dermatitis, pruritic dogs represent up to 30% of dogs presenting to the veterinarian for skin disease [4]. The anti-inflammatory effects of corticosteroids have led to their use in dogs when underlying allergic dermatitis is suspected, but they often have side effects [5, 6]. While corticosteroids have broad anti-inflammatory effects, many veterinary textbooks and peer-reviewed articles have noted the potential for long-term side effects especially when administered for greater than 14 days [1,2,3,5.6]. These side effects have been reported for most body systems [2]. Further, psycho-behavioral side effects have also been reported in dogs receiving prednisolone and methylprednisolone [5]. Many of these adverse effects focus on months or years of usage. However, the impact of short term (< 14 day) use is not as well studied and documented. As a frame of reference, a recent study in the UK using passive surveillance through reviewing medical records of dogs receiving corticosteroids, revealed an adverse event reporting rate of 4.9% [6], thus supporting the perception that few side effects are experienced by pets when short-term corticosteroids are used. However, the time frame for the UK study considered a prescription of up to 31 days of use [6].

In this study, pet owners were asked about the behavior and clinical signs of their dogs on day 5 and day 14 after starting anti-inflammatory doses of prednisone and prednisolone. Our aim was to use a survey tool to assess owner observed adverse events in dogs on anti-inflammatory doses of prednisone/prednisolone at 2 time points to determine the incidence of possible adverse events and whether longer treatment duration was associated with additional adverse events. We hypothesized dogs receiving anti-inflammatory doses of prednisone and prednisolone would experience much higher rates of side effects by day 14 than previously reported [6].

## Materials and methods

This research was conducted and reported in accordance with Strengthening the Reporting of Observational Studies in Epidemiology (STROBE) criteria [7]. All study participants were informed of the purpose of this study and written informed consent was obtained. Data from all pets and pet owners was anonymized and all legal requirements to protect this data have been adhered to. This study was reviewed and approved by the Zoetis Ethics Review Board and complies with the NIH guidelines for Humane Care and Use of Animals. All methods were carried out in accordance with relevant guidelines and regulations.

### Study Procedures

The study was conducted in 6 companion animal veterinary practices located throughout the United States. Pet dogs presenting to these practices with conditions where anti-inflammatory doses of corticosteroids were to be administered were considered for inclusion into the study.

#### Inclusion and Exclusion criteria

Generally healthy dogs of any age or weight could be enrolled into the study.

Dogs who were prescribed anti-inflammatory doses of oral prednisone or prednisolone for no less than 5 days were eligible to be included in the study. Investigators were given the choice to select doses of prednisone or prednisolone which they designated as treatment for inflammatory disease where immunomodulation was desired (e.g., allergic disease) and not immunosuppression (e.g., autoimmune disease) based on their real-world experience. Initial administration of the corticosteroid could be BID or SID and there could be dose reduction at the investigator’s discretion to SID or EOD. Tapering of the dosage included any reduction in the dosage over time and/or the frequency of administration. Any dog demonstrating clinical signs consistent with hyperadrenocorticism was excluded from the study.

All respondents provided informed consent prior to their participation. Study participants and investigators were compensated for their participation in the study.

### Data collection

**Fig. 1:**
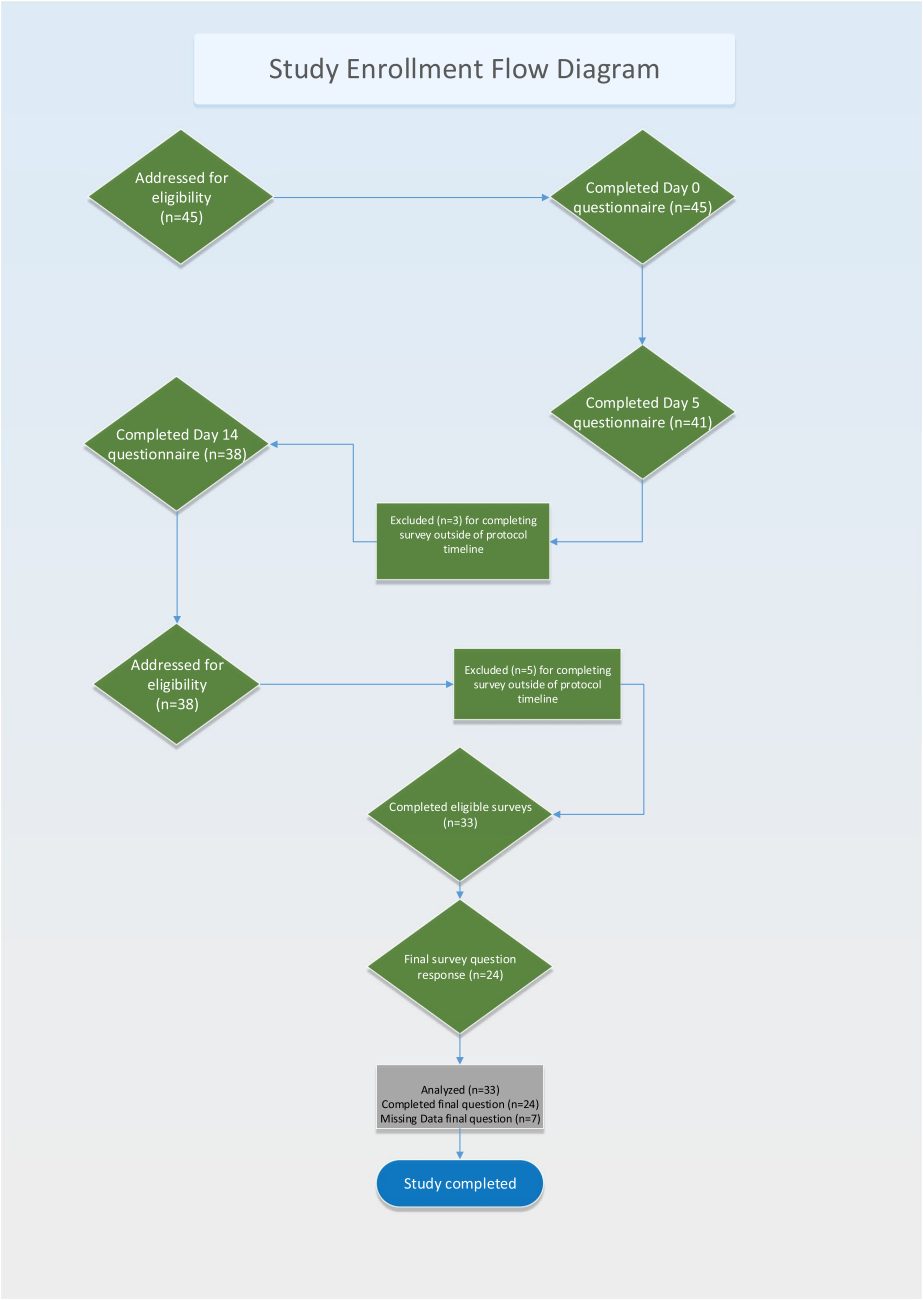
Study Enrollment Flow

Data collection occurred between January and December of 2020. The pet owner completed a paper survey on day 0 in the clinic at the initial examination prior to any corticosteroid administration. Surveys were sent electronically on day 5 (± 2 days) and day 14 (± 2 days) via text and email. All responses were gathered via the Qualtrics survey platform. Reminders were sent 24 and 48 h after the initial text/email to all respondents who did not complete the survey. A final single survey question was sent electronically via text to all respondents 30 days after they completed the day 14 survey. An additional reminder was sent via text 48 h later for any respondents who did not respond. Study enrollment is outlined in Fig. 1.

### Survey design

**Fig. 2:**
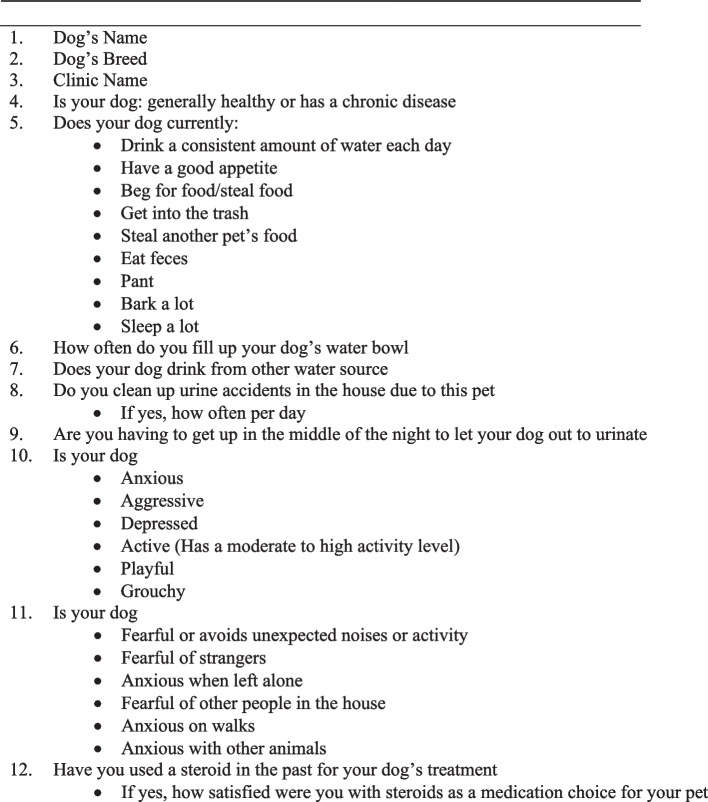
Day 0 Survey

**Fig. 3:**
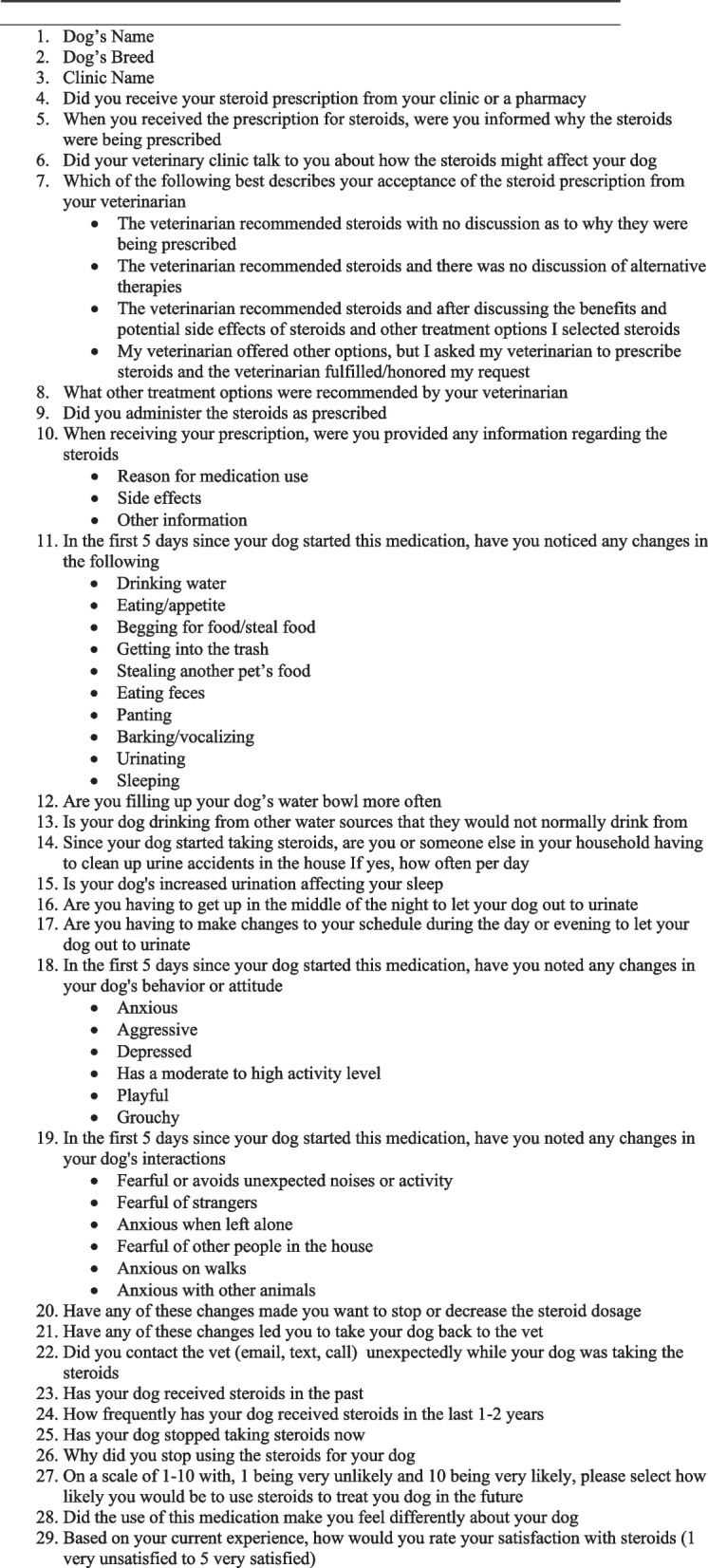
Day 5 Survey

**Fig. 4:**
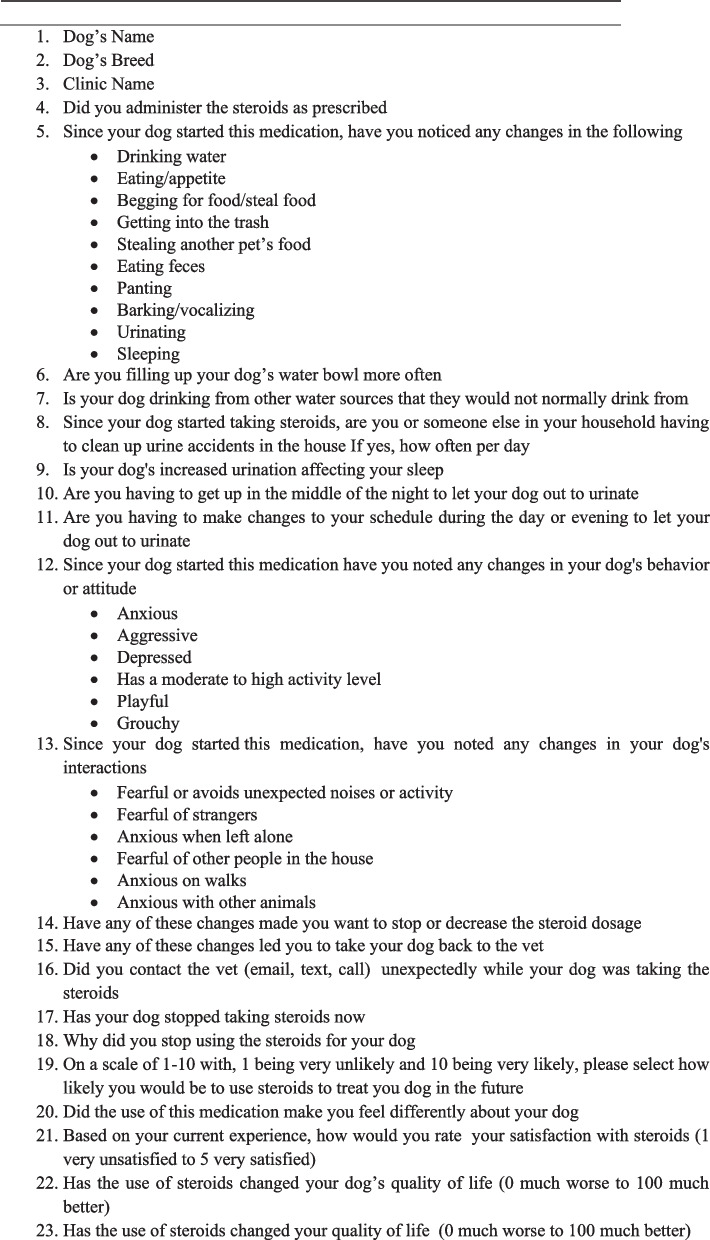
Day 14 Survey

The pet owner survey on day 0 was comprised of 10 questions designed to provide a baseline for each dog’s pre-treatment behavior and clinical signs (Fig. 2). A physical examination was completed by the veterinarian and an investigator form captured the reason for use, medication prescribed, dosage of prednisone/prednisolone, and treatment regimen. The day 5 survey was comprised of 37 questions designed to provide an understanding of changes occurring in the dog’s behavior (Fig. 3). In addition, pet owners were asked about their overall satisfaction with the use of prednisone/prednisolone. The day 14 survey was comprised of 29 questions designed to provide an understanding of the dog’s behaviors occurring by day 14 (Fig. 4). Logic was applied within the survey to remove follow up questions if not applicable based on a pet owners’ selection. Answers were not mandatory for survey questions and only 1 option could be selected for each question. For the activity section, pet owners were asked to consider the interval since starting prednisone/prednisolone and indicate if changes were greatly decreased, slightly decreased, no change, slightly increased or greatly increased compared to prior to administration of prednisone/prednisolone. For the behavior section, pet owners were asked to indicate if the behaviors were worse, no change, or better. Likert scoring was utilized with a range of 1–5 (1 = greatly decreased, 2 = slightly decreased, 3 = no change, 4 = slightly increased, 5 = greatly increased and 1 = worse, 2 = no change, 3 = better). For the activity and behavior sections, the definition of a slight versus great change was determined by the owner when comparing baseline to any assessed difference. Pet owners were also asked about their quality of life (QOL) as well as their pet’s QOL, which was completed only on day 14. On day 14, the study investigator was asked to provide information regarding communication (if any) which occurred between the pet owner and the clinic regarding the health and wellbeing of the dog while receiving the prednisone/prednisolone (beyond communications at day 5 and day 14). All behavioral study questions focused on behaviors previously found to change significantly in the presence of corticosteroid drugs [5]. The final question on day 30 focused only on the pet owner’s willingness to consider other therapies given their recent experience with prednisone/prednisolone.

To be included in the data analysis, pet owners had to complete the day 0 survey and at least one other (day 5 or day 14) post treatment survey. Pet owners who completed the surveys outside of the ± 2-day window or did not complete either a day 0 and day 5, or day 0 and day 14 survey, were excluded from analysis.

## Results

### Study population

There were initially 45 pet owners enrolled in the study. This included 4 pet owners who provided only day 0 responses and 8 pet owners who provided responses outside of the ± 2-day window resulting in 33 pet owners completing the study with evaluable results for either day 0 and day 5, or day 0 and day 14. The signalment of the dogs included in the study are summarized in Table 1.

**Table 1:** Pet signalment

**Sex**	**n**	**Age (y)**	**n**
Male	2	≤ 4	10
Neutered male	12	5–9	16
Female	2	≥ 10	6
Spayed female	17	Unknown	1

The most common reason for steroids to be prescribed was dermatitis or pyoderma (12 dogs), followed by otitis (11 dogs), flea allergy dermatitis (4 dogs), interdigital cyst (2 dogs) and 1 each for excessive licking, contact dermatitis, neck pain, or anal gland abscess. Thirteen pet owners (42%) had used steroids for their pet at least once in the prior 2 years.

Initial total daily oral doses ranged from 0.36 mg/kg to 2.64 mg/kg, with a mean starting dose of 0.79 mg/kg daily (Table 2). All of the dogs in the study received some tapering dosages (either number of doses per day or mg/kg per day). Initially 10/33 (30%) received BID dosing while the remainder started on SID dosing. Of the 10 dogs who received BID dosing, 5 received BID dosing for 3 days, 1 for 4 days and 4 dogs for 5 days. There were 17 dogs that had initial daily dosages which fell within the 0.5–1.0 mg/kg anti-inflammatory range reported in the literature [1]. However, seven dogs received a dose above the upper limits and 9 dogs received a dose below the lower limits of the anti-inflammatory range (Table 2). The mean duration of treatment was 14.6 days, with 15 dogs receiving a total of 10–11 days of steroids, 7 dogs receiving 10 days of treatment and 1 dog receiving a maximum of 28 days of treatment. On day 14, 16/31 (52%) of the dogs were still receiving prednisone/prednisolone with an average dose of 0.44 mg/kg (range 0.33 to 0.63 mg/kg) (Table 2). Ten of theses 16 dogs were on an every other day dosing protocol at day 14. Pets were grouped into small, medium, and large breeds and mean steroid dosing and ranges were calculated (Table 3).

**Table 2:** Individual dog age, weight, dose, regimen, and duration

Dog number	Dog Age (Years)	Dog weight (kgs)	Initial total daily dosage (mg/kg)	Tapering dosing protocol (Y/N)	Duration (Days)	BID to start (Y/N)	SID to EOD (Y/N)	Dose at Day 5 (mg/kg)	Dose at Day 14 (mg/kg)
1	8	28	0.36	Y	14	N	Y	0.36	0.36
2	5	39	0.51	Y	17	N	Y	0.51	0.51
3	1	35	0.56	Y	10	N	Y	0.56	N/A
4	11	23	0.43	Y	15	N	Y	0.43	0.43
5	10	6	0.85	Y	21	N	Y	0.85	0.42
6	11	22	0.90	Y	18	N	Y	0.90	0.40
7	15	8	0.65	Y	14	N	N	0.65	0.32
8	4	3	0.73	Y	14	N	N	0.73	0.36
9	13	4	0.63	Y	14	N	Y	0.63	0.63
10	12	6	0.78	Y	10	N	N	0.78	N/A
11	7	43	0.46	Y	10	N	Y	0.46	N/A
12	4	18	0.83	Y	14	N	Y	0.50	0.50
13	2	25	0.41	Y	12	N	Y	0.41	N/A
14	9	23	0.43	Y	12	Y	Y	0.43	N/A
15	7	87	0.46	Y	11	Y	Y	0.46	N/A
16	6	9	1.10	Y	11	Y	Y	1.10	N/A
17	4	28	1.10	Y	11	Y	Y	0.65	N/A
18	9	37	0.54	Y	21	N	Y	0.54	0.54
19	7	34	1.20	Y	20	Y	Y	1.20	0.60
20	5	11	2.64	Y	20	Y	N	2.64	0.44
21	2	34	0.88	Y	11	Y	Y	0.44	N/A
22	7	6	0.67	Y	11	Y	Y	0.34	N/A
23	8	17	0.87	Y	11	N	N	0.44	N/A
24	Unk	36	1.38	Y	11	N	Y	0.69	N/A
25	5	26	0.78	Y	11	N	Y	0.39	N/A
26	15	9	1.15	Y	20	N	N	1.15	0.57
27	4	3	1.45	Y	10	N	Y	0.78	N/A
28	6	43	0.47	Y	10	N	Y	0.24	N/A
29	2	16	0.94	Y	15	N	N	0.94	0.47
30	4	26	0.38	Y	10	N	Y	0.19	N/A
31	4	13	0.79	Y	28	Y	Y	0.79	0.39
32	6	29	0.69	Y	15	Y	Y	0.69	0.34
33	10	6	0.42	Y	20	N	Y	0.42	0.42

**Table 3:** Breed grouping, mean dose and dosing ranges

**Size based on Body weight**	**Distribution**	**Mean steroid dose**	**Dosage Range**
Small Breed	10	1.00 mg/kg	0.42 – 2.64 mg/kg
Medium Breed	10	0.68 mg/kg	0.38 –0.90 mg/kg
Large Breed	13	0.71 mg/kg	0.36 – 1.38 mg/kg

### Survey responses

Thirty-one pet owners completed the day 5 survey, and 31 pet owners completed the day 14 survey with 29 pet owners completing both day 5 and day 14 surveys, 2 pet owners completing only the day 5 survey and 2 pet owners completing only the day 14 survey. Tables include results for all 33 animals. All pet owners reported administering the steroids as prescribed.

### Day 5

#### Product dispensing and instructions

The majority, or 97% (30/31), of the pet owners had their prescriptions filled at the clinic, with 1 owner using a local human pharmacy. When considering communication, 90% (28/31) of pet owners reported their veterinarian discussed the reason for the use and potential side effects of corticosteroids with them at the appointment and all reported receiving verbal or written information regarding the reason the corticosteroid was dispensed. When asked specifically about side effects, 21 (68%) pet owners reported receiving information specific to the side effects of corticosteroids with 9 pet owners remembering only verbal information, 2 owners reporting only written information and 10 owners reporting a combination of verbal and written information. Side effect information, when written, was on a specific handout (5/31) or as part of their discharge instructions (6/31).

When considering how a decision to use prednisone/prednisolone for treatment was made, 74% (23/31) of the time veterinarians recommended prednisone/prednisolone but engaged with the pet owners in a discussion allowing the pet owner to make the final selection. For 10% of pet owners (3/31) the veterinarian initially recommended an alternative therapy instead of prednisone/prednisolone, but the pet owner specifically requested the use of prednisone/prednisolone and the veterinarian conceded. For the remaining 16% (5/31), the veterinarian made the selection of prednisone/prednisolone for the pet without discussion with the pet owner.

#### Pet owners reported changes in behavior

At day 5, 81% (27/31) of pet owners reported at least one change in their pet’s physical abilities (Fig. 3 question 11), behavior (Fig. 3 question 18) or demeanor (Fig. 3 question 19). There were 74% (23/31) of owners who reported at least 1 or more changes in their dog’s physical abilities including polyuria, polydipsia, polyphagia, polypnea and/or increased vocalization, with 11 individuals (35%) reporting these changes were greatly increased. Seventeen of 31 owners (55%) reported filling up the water bowl twice as often or more above baseline and 23% (7/31) reported having to clean up urinary accidents for dogs who were normally continent in the house. These behavioral changes were consistent across the weight ranges with 80% (8/10) small size dogs, 80% (8/10) medium size dogs and 77% (10/13) large breed dogs reporting changes on day 5.

Eight (26%) dogs had at least 1 behavior that owners reported as normal on day 0, but on day 5 had identified as worsened including anxiousness (2), aggressiveness (1), depression (2), or less active/playful (3) with 3 of these dogs also having their interactions with other dogs and people identified as “worse”.

For the 9 dogs receiving a prednisone/prednisolone dose below the lower limits of the anti-inflammatory range (< 0.5–1.0 mg/kg), 8 dogs had day 5 survey results. All 8 owners reported at least 1 change in their dog’s behavior at day 5.

Only 2 pet owners reached out to their vets during the first 5 days of treatment; 1 for clinical signs which did not resolve and the other after the dog developed diarrhea.

#### Pet owner satisfaction

At day 5, 97% of pet owners reported the prednisone/prednisolone controlled the condition for which it was prescribed. Pet owners reported general satisfaction with prednisone/prednisolone scoring a total of 3.87/5 in satisfaction scores. For pet owners who were neutral (5) or unsatisfied (3), 7 of the 8 owners (26% of our total population) reported polyuria and polydipsia, with 1 also reporting aggressiveness and 2 reporting negative behaviors with other dogs and people.

### Day 14

#### Pet owners experience with change in a pet’s behavior

On day 14, 28 owners (90%) reported at least 1 or more changes in their dog’s behavior including polyuria, polydipsia, polyphagia, and/or polypnea as the most common changes noted (Fig. 4 question 5). There were 61% (19/31) of owners who reported an increase in filling of the water bowl over day 0 and one-third (11/31) of pet owners were reporting cleaning up urinary accidents for pets who had been continent prior to the start of the study. By day 14, an additional 4 dogs had worsening in their behaviors when compared to day 5, resulting in 36% of the dogs in this sample size showing worsening of at least 1 behavior including anxiety, aggression and lethargy (Fig. 4 questions 12 and 13).

One dog owner discontinued medication between day 5 and day 14 due to aggressive and fearful changes in their dog’s behavior.

When dogs were grouped by age, there were no specific adverse events when comparing groups and the most commonly reported adverse events, polyuria and polydipsia were consistent across the groups (Tables 4 and 5).

**Table 4:** Adverse event by age group (Day 5)

**Age group (N)**	** < 4 (9)**	**5–9 (14)**	> 10 (6)	Unk. (2)
Polyuria	5	8	3	1
Polydypsia	5	8	2	1
Polypnea	3	4		
Polyphagia	3	7

**Table 5:** Adverse event by age group (Day 14)

**Age group (N)**	** < 4 (9)**	**5–9 (13)**	** > 10 (7)**	**Unk. (1)**
Polyuria	3	8	4	1
Polydipsia	7	7	5	1
Polypnea	3	7	4	1
Polyphagia	4	7	2	1

#### Pet owner satisfaction

Pet owners were asked to rate their overall satisfaction with steroids with a rating of 4.5/5 (1 = very unsatisfied, 5 = very satisfied). Comments reflected overall appreciation of improvement of clinical signs and reduction of the associated discomfort; for example, the dog felt better, the dog’s condition improved and life returned to normal.

#### Pet and pet owner Quality of Life (QOL)

On day 14, a total of 31 pet owners scored their dog’s QOL as a 73.6/100 and their QOL as a 55.9/100. For those pets who had urinary accidents, pet owners had a 30% lower QOL score (39/100) compared to the other pet owners in the study. For those pets who had an increase in aggression between day 5 and day 14, pet owners scored their dog’s QOL as a 65.9/100 and their QOL as a 51/100.

#### Lifestyle, activity and behavioral changes between Day 5 and Day 14

When considering both day 5 and day 14 results, 8 pet owners (27%) reported increases from slight to great in the behavior changes when comparing day 5 and day 14, and 20/31 (63%) owners reported new changes in their pet by day 14.

There was no additional veterinary clinic contact from any pet owner during the period between day 5 and day 14 of the study.

#### 30 day post study question

At the conclusion of the study, 23 pet owners completed a single final question with 70% (16/23) indicating they would select another product with fewer side effects even if the product cost more.

## Discussion

This study confirms the hypothesis that usage of prednisone or prednisolone changes the behaviour of the majority (81%) of dogs as soon as day 5 post-treatment. The behaviors reported in this study confirm previously reported changes noted in the presence of corticosteroid drugs [2, 5, 6] but at much earlier time points.

Glucocorticoids have been used for decades and remain one of the most prescribed medications in veterinary medicine [3, 8, 9]. Synthetic glucocorticoids like prednisone and prednisolone are considered to have an intermediate duration of action [1]. This intermediate action is based on the concept of biologic half-life which refers to the time for the effects (not plasma half-life) to abate by 50% [8]. For prednisone and prednisolone, this biologic half-life is 12 to 36 h [1]. This is one of the reasons these products are appropriate for every other day dosing. Anti-inflammatory dosages for dogs are recommended at a range of 0.5–1 mg/kg one to two times a day with a tapering dosing regimen [1, 8].

It is widely understood that use of synthetic corticosteroids like prednisone and prednisolone alter the production of corticotropin-releasing hormone (CRH) and adrenocorticotropic hormone (ACTH) which in turn change the hypothalamic–pituitary–adrenal axis [8]. This changes the normal secretory patterns of corticosteroids within the body and over time leads to adrenal atrophy [8]. It has been shown that even within 7 days of using anti-inflammatory doses (0.55 mg/kg daily) of prednisone, adrenal responsiveness to ACTH was suppressed [8]. Since glucocorticoid receptors are found in almost every cell type, exogenous steroid administration may affect single or multiple body systems [8].

Published guidelines identify the benefits of corticosteroids when used for treatment of canine atopic dermatitis [9–11] and note that the adverse effects of oral corticosteroids are normally proportional to drug dosage and duration of administration [10]. In this study, 39% of dogs received initial doses outside the anti-inflammatory range of 0.5–1.0 mg/kg, but side effects noted by the pet owners were reported regardless the of the dog’s initial dosage. In fact, for those dogs who received what are perceived to be lower than anti-inflammatory doses of prednisone/prednisolone, all reported behaviour changes at day 5. In addition, side effects noted by pet owners on day 5 were reported regardless of the pet’s size with all groups (small, medium and large) noting similar onset. Thus, these findings, while small in number, provide real data, which may draw into question the assumption of reduced side effects when administering a lower initial dosage.

While pet owners reported overall satisfaction with the use of prednisone/prednisolone, comments provided by the owners implied this satisfaction had less to do with the medication and more to do with their efficacy in reduction of clinical signs. It may be that as corticosteroids were discussed by the veterinarian with the owner, a clearer expectation was set, thus improving the pet owner's comfort with and acceptance of possible side-effects in their pet due to corticosteroid usage. With more recent medications available to the veterinarian [11], it is important to consider the primary driver for the satisfaction of the pet owner in this study was the overall improvement in clinical signs.

Only 2 (6%) owners contacted their veterinarian during the study period to discuss changes in their dog’s behaviour. This means 94% of our pet owners did not report any changes in their dog’s behaviour to their veterinary clinic, supporting the low incidence of adverse events reported in the UK VetCompass study [6], and underscoring one of the reasons veterinarians may underestimate the incidence of side effects when dogs are receiving prednisone/prednisolone.

In the UK VetCompass study, the median time to side effects after the administration of an oral glucocorticoid was 12.5 days and the most frequent side effects noted were polydipsia and polyuria [6]. Polyuria and polydipsia have been noted as the most consistently reported side effects [1, 2, 8–10]. In the UK study, dogs over 8 years of age were 4.24 times more likely to have polyuria and polydipsia than dogs 2 years of age or less [6]. While our sample size was notably smaller (4.9% (148/3000) vs. 90% (30/33)) we did not see this difference in our population with all the age groups showing overall consistence in numbers of adverse events. In addition, adverse events were consistent across the study population regardless of the initial dosing range or weight of the dogs.

During the study, veterinarians took time to inform pet owners of possible adverse events, however, this may not happen in busy clinics who are being impacted by so many other stressors. Any reduction to this level of communication may lead to an erosion of realistic pet owner expectation, increased interactions with the veterinary clinic, repeat visits, and potential dissatisfaction with the treatment selection for their pet.

Many owners were inconvenienced by their dog’s behavioral changes following the administration of prednisone/prednisolone with more than half having to fill water bowls more frequently and 1/3 having to clean up urinary accidents. It appears, based on the changes in QOL scoring for these owners, there is an increased burden of care that is added when a pet is impacted by side effects following the dispensing of medications like prednisone/prednisolone. To underscore this impact, 70% of pet owners were willing to accept the additional cost for a similarly effective treatment without side effects, indicating that while they were prepared for and accepting of these side effects, they would have preferred to avoid them and would have paid more for that benefit.

While veterinarians are familiar with the most commonly reported side effects of corticosteroids like polyuria and polydipsia, aggression and behavioral changes, while less common, may be particularly surprising and worrisome to pet owners. Pets displaying these reported behavior changes may put owners, family members, and other pets at increased risk for injury [5]. This study finding supports previously reported behavioral changes with corticosteroid use and adds additional importance to recommendations for behavioral risk management when products like prednisone/prednisolone are dispensed [5].

The limitations of this study include a small sample size and the potential bias associated with solicited pet owner reporting including the possibility of more stringent monitoring of perceived ADE’s. In addition, many of these pets had received steroids as treatment in the past and pet owners had their own expectations and comfort level regarding the prior treatment. We do not know how often steroids were offered to other pet owners but subsequently rejected, thus there is some self-selection bias for those who agreed to the use of corticosteroids that underlies our population. While this study represents real world usage, veterinarians were able to self-select “anti-inflammatory” doses of prednisone/prednisolone resulting in a much broader range of dosing than standardly defined. All investigators involved in the study were aware of the study and as such, were potentially more communicative regarding the nature and adverse event profile of the prescription, thus setting expectations for pet owners regarding the potential for adverse events and potentially biasing the owner’s level of satisfaction with the treatment and rate of adverse event reporting. Finally, pet owner and dog QOL was not assessed prior to the initiation of the study, thus the overall magnitude of impact of corticosteroid use and perceived adverse effects on the dog and pet owner QOL is not fully able to be appreciated.

Although the study sample was relatively small, several findings regarding short term prednisone and prednisolone usage are new findings not previously reported. Future work with larger populations would be recommended to understand if these same changes would be noted at a similar frequency.

## Conclusion

In this study, we hypothesized dogs receiving anti-inflammatory doses of prednisone and prednisolone would experience much higher rates of side effects by day 14 than previously reported. For the dogs enrolled in this study, prednisolone/prednisone resolved the inflammatory processes but were accompanied by common side effects in the majority of dogs. Even in dogs receiving lower than recommended anti-inflammatory dosing recommendations, onset of adverse events was noted as early as day 5. This study provides additional awareness of the early onset of adverse events noted when prednisolone/prednisone is administered to dogs.

## Data Availability

The datasets were obtained from a data sharing agreement and the data and analysis are the proprietary property of Zoetis, LLC.
